# Digital Health Policy and Cybersecurity Regulations Regarding Artificial Intelligence (AI) Implementation in Healthcare

**DOI:** 10.7759/cureus.80676

**Published:** 2025-03-16

**Authors:** Abdullah Virk, Safanah Alasmari, Deepkumar Patel, Karen Allison

**Affiliations:** 1 Department of Ophthalmology, Flaum Eye Institute, University of Rochester, Rochester, USA; 2 School of Health Sciences and Practice, New York Medical College, New York, USA; 3 Department of Public Health, School of Health Science and Practice, New York Medical College, Valhalla, USA

**Keywords:** ai and digital systems, ai governance, cybersecurity and healthcare, digital healthcare systems, healthcare disparities and digital health records, patient data security, racial and ethnicity bias. discrimination

## Abstract

The landscape of healthcare is rapidly changing with the increasing usage of machine and deep learning artificial intelligence and digital tools to assist in various sectors. This study aims to analyze the feasibility of the implementation of artificial intelligence (AI) models into healthcare systems. This review included English-language publications from databases such as SCOPUS, PubMed, and Google Scholar between 2000 and 2024. AI integration in healthcare systems will assist in large-scale dataset analysis, access to healthcare information, surgery data and simulation, and clinical decision-making in addition to many other healthcare services. However, with the reliance on AI, issues regarding medical liability, cybersecurity, and health disparities can form. This necessitates updates and transparency on health policy, AI training, and cybersecurity measures. To support the implementation of AI in healthcare, transparency regarding AI algorithm training and analytical approaches is key to allowing physicians to trust and make informed decisions about the applicability of AI results. Transparency will also allow healthcare systems to adapt appropriately, provide AI services, and create viable security measures. Furthermore, the increased diversity of data used in AI algorithm training will allow for greater generalizability of AI solutions in patient care. With the growth of AI usage and interaction with patient data, security measures and safeguards, such as system monitoring and cybersecurity training, should take precedence. Stricter digital policy and data protection guidelines will add additional layers of security for patient data. This collaboration will further bolster security measures amongst different regions and healthcare systems in addition to providing more means to innovative care. With the growing digitization of healthcare, advancing cybersecurity will allow effective and safe implementation of AI and other digital systems into healthcare and can improve the safety of patients and their personal health information.

## Introduction and background

The rapid advancement in the healthcare industry has been a catalyst for increased usage of many new forms of technologies in the field. Artificial intelligence (AI) is becoming more widely used in patient care through robotic surgeries, predictive models for diagnoses, and personalized treatment plans. In addition to the usage of digital tools in patient care, advanced devices and artificial intelligence have been used in many other sectors, such as research analysis, logistics, and data collection [1]. This increase in digitalization and usage of new technologies and devices in healthcare can pave the way for new cybersecurity threats. Especially with AI having access to large amounts of private health information, it could become a target for numerous cyberattacks, potentially jeopardizing patient safety. [1,2]. Therefore, it is critical to understand the implications of AI in order to properly implement it in a way that can avoid unnecessary cybersecurity risks while maximizing the productivity of the workforce and the benefits to the patients.

## Review

This review of literature included a comprehensive search of English-language publications from peer-reviewed journals in databases such as SCOPUS, PubMed, and Google Scholar between the years 2000 to 2024. Keywords used for the article search included artificial intelligence, healthcare, cybersecurity, liability, disparities, digital health policies, and ethics. Additional sources were also identified through the reference sections of the searched articles. Policy and news information was collected through government sites and reliable news organizations to discuss recent events and legislation. 

AI integration in healthcare

Artificial intelligence is used in various sectors of healthcare including medical research, disease diagnosis, health monitoring, and patient care. One of the most widely used applications of AI is diagnostic analysis for screenings in which it can help physicians analyze tests such as MRIs, X-rays, and CT scans in order to provide faster and more accurate diagnoses [1]. Machine learning, where the AI model can learn and improve from the data available, and deep learning, where AI can learn and continuously improve by itself through artificial neural networks, is used in many diagnostic settings to extract findings and abnormalities. AI algorithms designed for specific medical fields analyze large datasets to find patterns, abnormalities, and the eventual diagnosis [4,5]. In ophthalmology, deep learning has become a vital factor in analyzing ocular conditions such as diabetic retinopathy through large datasets and fundus photos [5]. In mammography, AI support helped radiologists improve their detection performance with increased sensitivity and specificity for cancer screening [6]. Other diagnostic uses of AI include predicting prognosis and therapeutic response based on histological findings in pathology, and improving clinical workflow. Companies are involved in the development of AI tools such as such as HALO™ (Indica Labs, New Mexico), Oncotopix® (Visiopharm, Denmark), and DeepLens (Amazon Web Services, USA) to detect and diagnose cancers, with many other initiatives being led throughout the various fields of medicine [7].

AI-driven robotics are also significant for surgical operations since these devices provide greater accuracy and faster recovery times. Robotic-assisted surgeries have made valuable contributions to many fields of medicine. In knee arthroplasty, robotic-assisted surgery had lower revision rates, lower systemic complications, lower opiate use for postoperative pain, and decreased occurrences of manipulation under anesthesia, which is a procedure to help regain motion if the knee replacement is stiff [8,9]. AI-powered robotic-assisted surgery is emerging as a significant advancement in medicine. With the extensive amount of global regulatory policies concerning data security and patient privacy, it is paramount to evaluate the applicability of all the regulations in assessing procedures such as cardiac surgery. An example is heart bypass operations performed with the Da Vinci robot, which allows for minimally invasive procedures with fewer complications [10]. Another usage of AI in medical care is through AI-powered chatbots such as Woebot or ChatPal, which allow instant access to medical information through natural language processing (NLP) [11,12]. This allows patients and clinicians to obtain quick access to medical information and recommendations, which can help reduce the workload for many physicians [1].

Artificial intelligence usage has also been implemented in patient monitoring as well with artificial information of things (AIoT) medical devices such as wearable health sensors. AI algorithms can constantly analyze vitals and compare with large amounts of health metric data to possibly predict future problems or signs of decline [5,13]. Machine learning techniques enable AI algorithms to also adapt to human behaviors in order to identify any abnormalities and provide more personalized monitoring.

Furthermore, AI use in medical research comes with many benefits. AI can analyze large data sets without using as much time and resources as manual analysis would usually take. AI can also identify certain patterns that would have gone unnoticed in addition to forming predictions and analyzing clinical relationships [1,14]. These predictive models can have large impacts on society, such as through disease spread models and contact tracing those who have been in exposed areas to inform people to quarantine, as seen in the COVID-19 pandemic [15]. Additionally, AI use has led to many advancements in genomics and drug discovery through large-scale predictive models and data analysis [1,14]. AI usage will continue to grow with the growing interest in analyzing large-scale data sets to acquire a deeper understanding of population health or certain questions that would require lengthy analysis.

Artificial intelligence models have also been shown to assist with cybersecurity by detecting phishing attacks [16]. With a majority of medical-based emails being automatically generated communication from the hospital or labs to confirm appointments or communicate results, increased amounts of cybercriminals are trying to replicate this form of communication in order to gain access to personal information. By understanding and detecting the patterns in phishing attacks or malware-infected emails, AI can collect data to find trends in phishing data and emails. From there, any future emails that have a similar pattern are classified and automatically filtered to spam to protect the individual and company data. Moreover, AI is able to proactively help with real-time monitoring of security risks and managing assets by determining vulnerabilities in the system and finding patterns or anomalies that indicate a security risk [17]. Antivirus or antimalware applications that are powered by AI software are also used in many industries to detect thousands of files to find malware. With regards to data protection, AI can monitor data movement and user activity to determine potential threats, violations of patient privacy and Health Insurance Portability and Accountability Act (HIPAA), and leaks based on intelligence data. Additionally, AI systems can quickly mitigate the spread of a virus or threat by isolating devices, which is especially useful in clinical sectors with large amounts of private health information. Further improvements can be recommended from security reports generated by AI algorithms, which will assist in any updates to security systems and protocols [17].

Digital health policies analysis

Cybersecurity regulations and health policies are crucial in providing a framework for the integration of AI and healthcare. Digital health policies are rules and regulations that address various technological integrations among healthcare which include telemedicine, health devices, and AI. For example, in the United States, HIPAA provided a revolutionary framework for protecting patient data and confidentiality but was passed before the widespread adoption of AI in healthcare [18]. While HIPAA focuses on individual identifiers, it may not adequately address issues pertaining to AI, such as the threat of re-identification of already anonymized data. This is only worsened when the data is handled by private entities due to poorer protection of privacy, risking the nature of patient confidentiality if data can be re-identified [19]. Still, these policies can be critical building blocks in the regulation of the increasingly AI-reliant healthcare system. Similar to HIPAA, the European Union General Data Protection Regulation (GDPR) also focuses on data protection and privacy but regulates AI partially by protecting individuals from solely automated decision-making and processing of health data unless consent was obtained [20]. However, regulatory bodies like the EU are proposing policies such as the AI Act that complement existing health policies in addition to building an advanced legal framework for the development and application of AI products [20]. By using existing policies as a framework, countries and institutions can create new revisions or policies that apply to the modern landscape of healthcare. Due to the rapid digitalization of various industries, there are different digital health policies around the world, each with varying strengths and limitations (Table 1) [21-26].

**Table 1:** Analysis of 5 existing digital health policies AI = Artificial Intelligence, IT = Information Technology, HIPAA = Health Insurance Portability and Accountability Act, HITECH Act = The Health Information Technology for Economic and Clinical Health Act, GDPR = The General Data Protection Regulation. [21-26]

Policy Name	Key Features	Strengths	Limitations
HIPAA	Privacy rule; security rule; enforcement rule	Strong patient data protection; clear guidelines for healthcare providers	No specific regulations for AI; no accountability for AI algorithms
HITECH Act	Promotes EHR adoption; strengthens HIPAA enforcement	Encourages adoption and advancement of health IT; improved patient data security protections	Emphasis on advancing EHR adoption; does not fully apply to AI
21st Century Cures Act	Interoperability promotion; prohibition of information blocking	Quicker innovations; enhanced data sharing	Potential privacy concerns; no specific regulations and recommendations for interoperability with AI
GDPR	Data minimization; purpose limitation; right to be forgotten	Strong data protection; broadly applicable to many sectors	Complex and costly compliance requirements; strict regulations can create a roadblock for AI development and data access
AI Act	Decrease risk exposure to high-risk AI; enforcement and obligations for AI developers	Stricter AI regulation; decreased privacy and security risks; increased transparency	Potential increase in compliance costs that can trickle down to consumers

With the plethora of global regulatory policies for healthcare data security and patient privacy already in place, it is critical to analyze the ability of those regulations to apply to AI in healthcare. There are many concerns regarding how these existing policies protect the privacy of patient data with large datasets being analyzed by AI algorithms. Major policies like HIPAA and GDPR provide a framework for protecting and managing patient data, but there is a significant need to address the challenges that are created with the use of AI algorithms to collect, sort, or analyze patient data. Furthermore, present and prospective opportunities encompass the integration of electronic health records among various health providers, the investment in health data science research, the generation of real-world data in a non-biased format including more diversity of collected data, the advancement of AI and robotics, and the promotion of public-private partnerships. Many ethical dilemmas and unforeseen repercussions arise from the implementation of any health information technology. To mitigate these issues, it is essential to establish regulatory frameworks governing the development, management, and procurement of AI and health information technology systems, foster public-private collaborations, and ensure the ethical and safe application of AI within the system to create a system based on science and trust [27].

A major priority for many countries around the world is improving the interoperability of artificial intelligence systems in healthcare while still upholding security standards that are used by the industry. The prospect of a more productive healthcare environment with improved quality of care is a prime reason for the rapid implementation of AI. However, regulations must achieve a balance that protects patient health information while allowing for innovation in the AI industry [28]. This remains difficult as it is largely ignored in many digital health policies.

Along with interoperability, many countries have issues with the transparency of certain policies when it comes to AI and the liability surrounding it in healthcare. Regulations about liability tend to be vague when it comes to AI systems’ accountability, resulting in inconsistent case law [29]. This lack of transparency has the potential to lose the trust of healthcare professionals regarding AI-generated advice. With the AI ecosystem containing multiple parties involved, such as clinicians, healthcare systems, and manufacturers, it is vital that the consequences fall upon those who are actually liable. Physicians or healthcare systems face liability for malpractice and other negligence while the manufacturers or designers of the AI tool might face product liability. For example, if the physician interprets the AI output incorrectly, deviates from the standard of care, or uses it on the wrong patient group, the liability will fall on the clinician [29]. On the other hand, developers would be liable for injuries that resulted from errors in the product. The standards for healthcare software products have been inconsistent and have put more risk of liability on the physicians and healthcare systems [29]. In addition, with many AI algorithms lacking explainability, it is difficult for physicians to accurately assess the viability of the results produced by the algorithm, which further increases the difficulty in placing liability [30]. Many clinicians are not adequately trained, and patients are not adequately educated. However, liability falls on clinicians, while the outcome affects the patient. It is evident that the modern status quo is not a very equitable system and more efforts at educating each party should be done. There is much room for improvement regarding digital health policy and cybersecurity regulations, and it is critical that regulatory bodies dedicate extensive resources to create clear and adequate guidelines.

Cybersecurity risks for healthcare data

With examples of large-scale cybersecurity and technological systems in healthcare facing challenges due to cyberattacks or even simple updates that are caused by unintended bugs, lack of testing, and memory leaks, the healthcare system faces many challenges that threaten both the privacy and health of the patients. In the case of the 2024 cyberattack on Change Healthcare, which was likely due to a lack of multi-factor authentication, millions of health records and patient private health information were put at risk [31]. In addition to the attack costing Change Healthcare an estimated yearly cost of around 1.6 billion dollars, the disruption of the payment and claims processing due to the attack financially hurt various healthcare systems and individual practices around the US [32]. On the other hand, a simple software update in 2024 by CrowdStrike, a cybersecurity firm, led to a major disruption in healthcare systems amongst many other sectors by preventing Windows devices from working. As a result, health records were not able to be accessed, ID badges were not working, and emergency systems were down, which impacted both the practices and the health of the patients [33]. Current cybersecurity challenges in healthcare include protecting electronic health records, securing medical devices, and preventing ransomware attacks. Many countries around the world utilize electronic medical records, payment systems, and online communication, resulting in an increased threat of data leakage or theft. With the heavily digitized international healthcare industry today, advanced cybersecurity measures are needed to ensure the protection of patient data and the functionality of healthcare practices. As AI utilization increases in healthcare, the risk to patient data will only rise unless proper measures are put in place. On the other hand, utilization of AI in cybersecurity is not inherently bad as it can rapidly adapt to cyber threats. AI’s role in cybersecurity assists in protecting patient data and creates a duality of purpose. In the future, finding the right balance and the best methods for AI usage will be essential for patient safety.

Cybersecurity implications of AI in healthcare

The implementation of AI in the US healthcare industry is projected to increase, with the AI market in healthcare predicted to have a compound annual growth rate of 44.2% between 2019 and 2027 [34]. With the rapid digitalization and implementation of AI in healthcare, many cybersecurity vulnerabilities have been introduced. With the large amount of patient data that AI systems have access to, serious cybersecurity risks of data breaches or leaks can arise [1]. As AI becomes more prevalent in the industry, a safeguard that organizations should implement is to minimize healthcare data exposure to AI systems, which can reduce the amount of patient data at risk in case of a data breach. However, since AI systems require extensive data access for algorithm training, developers and countries have to balance AI innovation with patient privacy in order to foster advances in the industry [28]. Given the recent cybersecurity events in healthcare that highlight the lack of proper cybersecurity checkpoints, AI handling of large amounts of data will only provide increased possibilities for hackers to detect vulnerabilities and gain exposure to sensitive medical information and patient records. An alternative that organizations can use is to allow AI systems to generate synthetic data for the training of its models. However, this, in turn, raises concerns about the diversity and effectiveness of the training itself. It is essential that organizations develop certain guidelines and standards that achieve a balance of both innovation and data security.

Artificial intelligence of things (AIoT) medical devices that use AI, such as smart health monitors and smartwatches, to collect and store patient health data in order to anticipate potential problems and needs. However, these devices have also been known to be vulnerable to security threats [2,13,35]. Cyberattacks and hackers can cause impaired device functionality in addition to leakage of health information and harm to the patient’s health. On the other hand, Yamin et al highlighted that AI can be used on the offensive by breaching and manipulating medical records for financial, malicious, or political purposes [16]. AI can help cyberattackers adapt to detection systems by obtaining new data, thus allowing a potential evasion of security systems. This problem is particularly growing in importance as an official FBI notice warned about the threat of cyber criminals using AI in social engineering/phishing attacks by using artificially manipulated audio and visual content [36].

Impact of AI on health disparities

With the implementation of AI models into healthcare, it is becoming increasingly necessary to highlight the importance of diversity in AI. Especially with the sheer diversity in healthcare patient populations, it is imperative for AI systems to be trained in diversity and inclusion. If AI training does not include diversity, biases and lack of fairness could emerge during patient care which risks generalizability in AI healthcare decision-making [37,38]. With over half of the databases used to train AI-based models coming from the US or China, other populations who are not included in AI training are left at a disadvantage, thus amplifying healthcare disparities that were already present [37]. In addition to gathering data on different populations, datasets and studies need to be collected through various institutions in different regions to prevent the systemic bias of individual providers and healthcare systems from impacting the decision-making of the AI model [39]. Hence, it is necessary for AI companies to disclose how the models were trained to allow physicians and other clinicians to make accurate assessments of the applicability of the model in a certain patient population. Increased interpretability of AI models and training for healthcare providers will make identifying bias in algorithms easier [39]. Especially in cases with solo practice physicians in rural areas that serve minority communities, the understandability of the implicit biases that AI algorithms carry is necessary to prevent incorrect diagnoses and unnecessary costs to both the provider and patient. Eduard Fosch-Villaronga et al emphasizes the need for diversity in AI for medicine with the potential impact that algorithm bias may have on discrimination, safety, and privacy concerns for patients in increasing automated medicine. The current algorithm-based systems may lead to bias and, therefore, may provide misdiagnosis and miscalculations in racial minorities and marginalized communities [40]. They also highlighted that extending AI technologies that do not account for diversity risks unsafe and inadequate healthcare delivery. Development and implementation of AI algorithms in an inclusive diverse population will avoid exacerbations already existing in our system, and the formation of new prejudices [40]. Moreover, when using AI to guide studies, clinical trials, and treatment designs, it is essential to intentionally have diverse participants with respect to race, ethnicity, age, and gender to obtain inclusive and unbiased results [40]. In doing this, we have to ensure that all participants from different backgrounds are included in an appropriate number that is representative of their respective population proportions. Gender, sex, race, and ethnicity are used in AI algorithms in the healthcare context, research, and patient care; thus, they need to be leveraged to decrease bias and have more inclusive results. Consequently, interventions and solutions created by AI algorithms will be able to generalize to people of different races, ages, and genders, resulting in better overall outcomes.

Current landscape of healthcare cybersecurity

The United States and other western countries have been major players in the global healthcare cybersecurity market, with companies such as IBM, McAfee, Cisco Systems Inc., and Broadcom Inc. However, regions such as Asia will include some of the fastest-growing markets for healthcare cybersecurity with many countries having rapidly growing IT sectors and increased risk of security breaches in healthcare systems [41]. Countries like China, Japan, and India are making headway towards the digitization of healthcare [3]. As global healthcare reliance on technology and internet-based systems continues to grow, it is essential to promote collaboration between markets in different regions. As a result, preventable security breaches and challenges can be avoided while also enabling a venue for other regions to create their own sectors. Collaborative research and development initiatives can pave the way for innovation in cybersecurity solutions and allow for increased diversity in AI training algorithms.

True cost of cybersecurity

As global digitalization continues to increase, cybercrime is rapidly growing in prevalence around the world. Estimates have reported that cybersecurity threats cost the world roughly 6 trillion US dollars in 2021 [42,43]. This cybercrime cost is especially rising in healthcare, as cybercriminals use the confidential nature of healthcare data to gain ransom money from organizations or sell it to vendors. With over 42 million US patients being affected by cybercrime from 2016 to 2021 and the healthcare system in the US incurring a loss of 6 billion dollars in 2019, there is a growing need to invest in advanced cybersecurity measures [44,45]. Hence, it is critical for healthcare organizations to adhere to strict cybersecurity guidelines by conducting internal audits, prioritizing training programs, and innovating existing cybersecurity measures [45]. However, funding is a major issue for many organizations, hospital systems, and private entities, such as multi-specialty and solo practices, especially given the high cost for cybersecurity professionals, insurance policies, systems, and organization-wide training. Therefore, governments need to be involved and provide the necessary funds to protect patient data, provide funding for organizational implementation, and prevent any unnecessary financial losses due to cybercrime.

Recommendations

It is crucial for healthcare organizations and policymakers to address the issues that tag along with increased AI implementation in healthcare. The current legal framework and security structure are not enough to sufficiently address the various risks. As machine learning and AI algorithms collect and analyze personal data, there is an increased risk of security breaches [46]. With this increasing risk, more proactive strategies must take place to prevent significant jeopardization of patient data and safety. An effective strategy is to restrict AI to the least amount of patient data it needs to have adequate training and to make effective clinical decisions while also de-identifying the data in case of a data breach. However, while protecting patient privacy is a necessity, it is important not to apply too many restrictions that would lead to stunted innovation in the field or inadequate and ungeneralizable training of AI algorithms [47]. Other strategies could include creating real-time records of anyone interacting with patient records and requiring training of all staff in cybersecurity in hospital systems. With a majority of healthcare data breaches coming internally due to lack of training and negligence, employee training and education is a necessary and vital step toward protecting patient data [48]. By ensuring proper training and certification for all employees to reduce the risk of human-caused data leaks, healthcare systems can dedicate more resources toward advancing the cybersecurity of AI algorithms. Policymakers should clearly identify the liability across the various sectors to appropriately allocate responsibility when it comes to artificial intelligence [30]. Healthcare policies should be transparent and should not be shifting the majority of the burden on the physicians, but equally placing it amongst the various actors related to AI. This clarity fosters the appropriate application of AI and lessens the legal ambiguity (Table 2).

**Table 2:** Recommendations for AI Implementation into Healthcare. AI=Artificial Intelligence [49-50]

Recommendations	Description	Potential Impact	Implementation Challenges
Require transparency with AI and other applications	Requiring AI developers to disclose the development of the algorithm, source codes, data inputs, and analytical approaches to its vendors or ethics committees.	Improved trust in AI systems in addition to easier assessments by hospital systems.	Private developers might not want the release of intellectual property. Potential government systems are a valid solution to this.
Government data protection standards and laws	Stricter data protection guidelines, such as access restrictions, will add another layer of safety for patient data.	Safer AI systems in hospital networks; ability to blacklist/fine AI systems that do not meet ethical requirements and pose a risk to patient safety.	Data protection authorities or AI developers might show some resistance.
Continuous AI system vulnerability assessments	Implement ongoing system monitoring and health checks that will identify any weaknesses or abnormalities.	Continuous system monitoring can determine and identify faults and prevent future breaches.	Can be very resource-intensive, and might need additional funding to properly implement.
AI ethics board requirement	Mandate that all hospital systems that use AI to a certain level must have an ethics committee.	This helps with answering any question regarding ethics in addition to adding another layer of protection to patient data.	Will require training for committee members in addition to additional funding.
AI-specific liability framework	Concrete standards meant to clearly appoint liability to certain parties in cases of AI system errors or breaches. Clear responsibility of burden of proof on a party for the situation.	This allows for most trust in AI in addition to a more just legal framework to appoint responsibility for certain faults.	Could be an initial challenge to properly define parameters for liability.
Mandatory AI cybersecurity training and awareness programs	Require cybersecurity training for all healthcare staff to specifically teach about the security risks of AI.	Improved awareness of security risks will help protect the whole hospital system from any breaches caused by individuals.	This method would be very resource-intensive, and might have to be merged with other training modules.

Additionally, rules should encourage the sharing of knowledge about cybersecurity threats and best practices across healthcare companies. In the healthcare industry, cooperation and knowledge sharing can strengthen the group’s security against cyberattacks [51]. The sharing of data, such as healthcare charges, can allow easier detection of fraud, which further saves costs [52,53]. Moreover, frameworks such as the one launched by the US National Institute of Standards and Technology, which promotes a collaboration between the US government and private entities to improve cybersecurity, are a necessary step towards protecting patient data. Collaboration would allow the sharing of resources and strategies to optimize cybersecurity and also improve patient and physician trust in the technology, which would in turn benefit the healthcare system as a whole due to its lack of resources in IT [48]. Another method to promote the sharing of knowledge is the consolidation of healthcare insurance companies. This can be seen in countries with single-payer or multi-payer universal healthcare, which allows for consolidation of health insurance into a single unit or multiple providers respectively [53,54]. In addition to saving in administration costs through consolidation of billing, fewer insurance payouts, and the price negotiation, the single-payer model of insurance will allow better care for the population through increased access and economical clinical services and administrative costs. By allowing easier access to healthcare screenings and prevention programs, the long-term costs can be lessened by reducing incidences of diseases [53,54]. Single-payer systems are usually controlled by the government which regulates healthcare spending through government funds. However, complete consolidation could lead to potential monopolistic control of the market if the single-payer misuses the power. There is no correct answer to this dilemma as different countries have varying situations. This is seen with many countries that provide universal healthcare choosing a more complex and costly multi-payer system that also involves private entities, which in turn increases competition for innovation [53,55]. Nevertheless, consolidation and collaboration to some degree in health insurance will benefit through sharing information about both cybersecurity and healthcare indicators, which will allow for more innovation and advancement of the industry [53,54]. Finally, in order to maintain patient safety and trust, we need a diverse AI algorithm development that is accounts for race, ethnicity, gender, and age. Diverse patient populations that are used in AI training increased ability for AI guidance to be safely applicable to a diverse range of people.

## Conclusions

With the evolving nature of the healthcare industry, more advanced technologies and forms of artificial intelligence will continue to make their impact. However, many cybersecurity risks will emerge with the increased implementation of new technologies and AI models. Thus, governments must take the initiative to pass health policies that factor in this new wave of advancement. Healthcare organizations also need to be proactive in promoting employee awareness in addition to developing strategies to prevent any serious issues and protect sensitive patient data. It is also important to establish clear liability parameters for defining the responsibility in case of adverse cybersecurity outcomes caused due to AI integration. As artificial intelligence continues to integrate into healthcare, inclusivity in AI training and data collection by incorporating diverse populations in algorithm training should be of importance to both the providers and developers to allow for adequate generalization to diverse patient populations. In order to foster patient education and safety, developers and companies should maintain transparency of AI algorithm training and tendencies to the providers. This will allow clinicians to make accurate assessments and better assist patients in making more informed decisions. With the growing prevalence of AI and digital innovation in healthcare, the importance of cybersecurity should take precedence to improve the safety of patient data, accuracy and diversity of data, implications in patient care of a diverse population, and the trust of patients, physicians, and all healthcare workers in the system. Integrating the results will lead to increased access, enhanced quality, and equity of care as well as reducing the cost of care. 
